# The Value of Soluble Urokinase Plasminogen Activator Receptor (suPAR) as Predictive Tool in Hospitalised Patients With Community‐Acquired Pneumonia (CAP)

**DOI:** 10.1111/crj.70089

**Published:** 2025-06-16

**Authors:** Lisa Hessels, Ruud Duijkers, Marianne Schoorl, Lotte Terpstra, Willemien Thijs, Wim Boersma

**Affiliations:** ^1^ Department of Respiratory Medicine Northwest Clinics Alkmaar the Netherlands; ^2^ Department of Respiratory Medicine Medical Center Leeuwarden Leeuwarden the Netherlands; ^3^ Department of Clinical Chemistry, Haematology & Immunology Northwest Clinics Alkmaar the Netherlands

**Keywords:** biomarkers, pneumonia, prognosis, severity

## Abstract

**Background:**

Soluble urokinase plasminogen activator receptor (suPAR) is a biomarker elevated in severely ill patients, but its prognostic value in community‐acquired pneumonia (CAP) remains unclear. This study aimed to evaluate suPAR's prognostic role for CAP severity compared to other biomarkers and severity scores.

**Methods:**

A total of 204 hospitalised CAP patients were enrolled. C‐reactive protein (CRP), procalcitonin (PCT), suPAR, CURB‐65 and Pneumonia Severity Index (PSI) scores were measured at admission, and patients were followed for 365 days. The primary outcome was the relationship between suPAR levels and CAP severity based on IDSA/ATS guidelines. Secondary outcomes included time to clinical stability (TTCS), length of stay (LOS) and mortality.

**Results:**

Among 204 patients, 174 (85%) had non‐severe and 30 (15%) had severe CAP. SuPAR levels were not associated with severe CAP (OR 1.03, 95% CI 0.88–1.21; AUC 0.53). Unlike the PSI and CURB‐65 scores, suPAR did not correlate with TTCS (HR PSI 0.80, HR CURB‐65 0.86), though all three markers were correlated to LOS (AUC suPAR 0.61). Only suPAR was significantly associated with 30‐day mortality (HR 1.51, AUC 0.68).

**Conclusions:**

The prognostic value of suPAR for CAP severity is low, and it does not provide additional prognostic benefit over the CURB‐65 or PSI scores in predicting CAP severity. While it has moderate predictive ability for 30‐day mortality, its utility for predicting LOS or TTCS is low.

**Trial Registration:**

ClinicalTrials.gov identifier: NCT01964495

## Graphical abstract

This study evaluated 204 hospitalised CAP patients, measuring suPAR, CRP, PCT, CURB‐65 and PSI at admission, with outcomes tracked over 365 days. SuPAR showed limited value in predicting CAP severity compared to CURB‐65 and PSI.

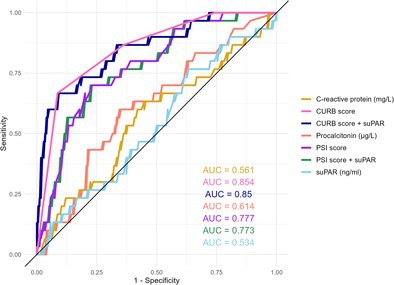

## 1 Introduction

Community acquired pneumonia (CAP) is a common infection in the Western world, with an estimated incidence between 5 and 11 cases per 1000 persons per year [1]. Furthermore, CAP is an important cause of mortality, especially in elderly patients [2, 3, 4]. Accurate risk stratification of CAP patients is necessary for guiding therapeutic decisions and allocating healthcare resources.

Conventional clinical scoring tools have limitations that underscore the need for better prognostic indicators. The most widely used and validated clinical scoring tools in CAP are the Pneumonia Severity Index (PSI) score and the CURB‐65 score [5, 6, 7]. However, the CURB‐65 score only contains a few items, thereby reducing specificity. The PSI score is more extensive and therefore less used in clinical practice. Both tools also contain items that are dependent on individual interpretation.

In addition to clinical scoring systems, various biomarkers have been studied for prognostic prediction in CAP. Two biomarkers that are often used in clinical settings are C‐reactive protein (CRP) and procalcitonin (PCT). The use of these biomarkers accelerated during the recent COVID‐19 pandemic. However, previous studies have shown that CRP and PCT are predominantly useful for diagnosis and monitoring of treatment and less useful for determining severity and prognosis of CAP [8, 9]. Thus, other biomarkers are needed to provide additional prognostic information alongside the clinical scoring systems.

A candidate as a prognostic biomarker in CAP is soluble urokinase‐type plasminogen activator receptor (suPAR). SuPAR is a soluble form derived from the proteolytic cleavage of urokinase‐type plasminogen activator receptor (uPAR), a membrane protein expressed on various cell types, including immune cells, endothelial cells and malignant cells. The urokinase plasminogen activator system plays a role in tissue remodelling, inflammation and tumourigenesis [10, 11, 12, 13]. SuPAR's release during inflammation or immune activation, coupled with its non‐specific nature, makes it a potentially informative marker for assessing the progression of infectious and inflammatory disorders [14]. Previous studies have shown that suPAR has a prognostic value in patients with ARDS and sepsis [15, 16, 17]. Furthermore, two previous studies indicated that suPAR might also have prognostic value in patients with pneumonia [18, 19]. Noteworthy is that suPAR has been used in an algorithm of COVID‐19 treatment, where it has demonstrated predictive capabilities for respiratory failure, kidney injury and overall clinical outcomes. The SAVE‐MORE trial used a cut‐off of suPAR > 6 ng/mL to start treatment with anakinra in patients with COVID‐19 [20]. It is still unclear whether this cut‐off can also be used in patients with CAP.

In the present study, we aimed to determine the diagnostic value of suPAR to predict the severity of CAP.

## 2 Methods

### 2.1 Study Design

This study is a sub‐analysis of patients included in a multicentre randomised controlled trial involving patients hospitalised with radiologically proven CAP from three hospitals in the Netherlands. For this sub‐analysis, only patients from the Northwest Clinics were included. The study protocol was approved by the Medical Ethics Committee associated with Northwest Clinics and is in full compliance with the Helsinki declaration. Recruitment of participants started in December 2013 and was completed in December 2016.

### 2.2 Participants

All adult patients with a clinical diagnosis of CAP were assessed for eligibility. Patients were eligible for inclusion in the study if they met the following inclusion criteria: age ≥ 18 years, required hospitalisation and had an estimated life expectancy of > 30 days based on clinical judgment at admission. This criterion was included to exclude patients with a very poor baseline prognosis to ensure meaningful follow‐up. All patients had a new infiltrate on chest X‐ray and the presence of one or more of the following signs and symptoms: temperature ≥ 38°C, dyspnoea, cough, chest pain, malaise or fatigue, gastrointestinal symptoms, rales/rhonchi or wheezing, egophony or bronchial breath sounds and haemoptysis.

Exclusion criteria were severe immunosuppression as judged by the investigator (e.g., HIV infection, chemotherapy and immunosuppressive drugs with exclusion of low‐dose corticosteroids), active neoplastic disease, obstructive pneumonia, aspiration pneumonia, pneumonia that developed within eight days of hospital discharge, expected inability to comprehend or follow the study protocol, pregnancy, lactation and unable to give informed consent (either patient or legal representative).

At the time of admission, patients gave consent for drawing an extra blood sample, and after 2–3 days, they were asked to participate in the study and gave full written informed consent. If another diagnosis was established prior to randomisation and antibiotic treatment was stopped, patients were not randomised and excluded from the study.

### 2.3 Laboratory Testing

Serum samples were collected on the day of hospitalisation and were stored at −80°C until analysis. Serum suPAR levels were measured using an enzyme‐linked immunosorbent assay (ELISA) kit (suPARnostic AUTO flex ELISA, Virogates, Copenhagen, Denmark). To check the validity of the suPAR levels of participants, suPAR was also measured in the serum of 12 healthy participants. CRP and PCT measurements were also determined on the first day of admission.

### 2.4 Data Collection

All data were prospectively collected in a standardised manner. Baseline assessments included clinical data and vital signs, comorbidities according to the Charlson Comorbidity Index, smoking status, medication and routine laboratory tests. Chest X‐rays were reviewed by attending physicians and/or radiologists. CURB‐65 scores and PSI scores were calculated. Blood cultures, urinary antigen tests for Pneumococci and Legionella and an oropharyngeal swab for respiratory viruses and atypical pathogens were collected. If possible, sputum cultures were obtained for all patients. All outcomes were assessed at an outpatient visit at day 30 ± 2.

### 2.5 Outcome Measurements

The primary outcome was severe CAP, defined as the presence of at least one major criterion or at least three minor criteria, according to the 2007 IDSA/ATS guideline [21]. Major criteria included: (1) requirement for invasive mechanical ventilation and (2) requirement of vasopressors for septic shock. Minor criteria included: (1) respiratory rate ≥ 30 breaths/min; (2) oxygenation index (PaO_2_/FiO_2_) ≤ 250; (3) presence of multilobar infiltrates; (4) presence of confusion or disorientation; (5) uraemia (blood urea nitrogen ≥ 20 mg/dL); (6) leucopenia (white blood cell count ≤ 4 × 10^9^/L); (7) thrombocytopenia (blood platelet count < 100 × 10^9^/L); (8) hypothermia (core temperature < 36.0°C); and (9) hypotension requiring aggressive fluid resuscitation.

Secondary outcomes were time to clinical stability, 30‐day mortality and hospital length of stay.

Clinical stability was defined according to Halm's criteria [22]: Temperature ≤ 37.8°C, heart rate ≤ 100 beats/min, respiratory rate ≤ 24 breaths/min, systolic blood pressure ≥ 90 mmHg, arterial oxygen saturation ≥ 90% or pO_2_ ≥ 60 mmHg on room air, ability to maintain oral intake and normal mental status.

### 2.6 Statistical Analysis

Given that this study is an exploratory analysis within a subset of the original trial population, no additional sample size calculation was performed specifically for this sub‐analysis. Variables were described using standard descriptive statistics. Comparisons between two groups were performed using the Wilcoxon rank sum test. Categorical variables were compared using the chi‐squared test or Fisher's exact test. Correlations were analysed using Spearman's rank test.

Univariate logistic regression models were used to compare the association between suPAR levels, the CURB‐65 score and the PSI score, CRP, PCT and bivariate outcomes. To test the added value of suPAR levels in addition to the CURB‐65 score and PSI score, a multivariate logistic regression model was used. The difference between the univariate and the composite models was tested with a likelihood‐ratio test. If the likelihood‐ratio test indicated added value, the capacity of the models with and without suPAR for risk reclassification was compared using the net reclassification improvement (NRI) and the integrated discrimination index (IDI). The discriminative capacities of the models were shown using the area under the curve (AUC) of the receiver operating characteristic curve (ROC).

Cox proportional hazards models were used to compare the association between SUPAR levels, CURB‐65 score, PSI score, CRP, PCT and time‐dependent outcomes. Predictive accuracy was calculated for clinical stability by day 7. Predictive accuracy for mortality was calculated at day 30. Predictive accuracy for length of stay was calculated for day 7. The discriminative capacities of the models were shown using time‐dependent ROC curves, and the discriminative capability was calculated using Uno's inverse‐probability‐of‐censoring weighting (IPCW) area under the curve (AUC). Cox proportional hazards assumptions were checked for each predictor.

Each model was submitted to internal validation by bootstrapping, and performance indices corrected for optimism were calculated. Calibration of the models was assessed with a calibration plot. All analyses were performed with R Statistical Software (version 4.2.1, R Core Team 2021). A *p*‐value < 0.05 was considered statistically significant.

## 3 Results

### 3.1 Baseline Characteristics

Samples for additional analysis of suPAR at admission were available from 204 of 255 enrolled patients. The baseline characteristics of the included patients are presented in Table 1. Of the enrolled patients, 174 (85%) were classified as non‐severe CAP, and 30 (15%) were classified as severe CAP. Differences in baseline characteristics in the non‐severe CAP patients versus severe CAP patients are displayed in Table 1. No significant difference in suPAR levels between the non‐severe CAP and severe CAP groups (3.9 ng/mL vs. 4.0 ng/mL, *p* = 0.60) was demonstrated. However, PCT was significantly higher in the severe CAP group (2.4 μg/L vs. 0.5 μg/L, *p* < 0.047), whereas WBC and CRP levels were not significantly different between both groups.

**TABLE 1:** Baseline characteristics of patients with CAP.

	Characteristic	Overall, *N* = 204	Non‐severe CAP, *N* = 174	Severe CAP, *N* = 30	*p*‐value^a^
Age		69.0 (60.0, 80.0)	68.0 (59.3, 78.0)	77.0 (68.3, 84.8)	0.004
Sex (male)		124 (61%)	98 (56%)	26 (87%)	0.002
Smoking status	Former smoker	112 (58%)	94 (57%)	18 (64%)	0.4
	Current smoker	51 (26%)	43 (26%)	8 (29%)	
Antibiotic pre‐treatment		44 (22%)	40 (24%)	4 (14%)	0.2
Comorbidities	Congestive heart failure	21 (10%)	16 (9.2%)	5 (17%)	0.2
	COPD	74 (36%)	56 (32%)	18 (60%)	0.003
	Chronic renal disease	10 (4.9%)	8 (4.6%)	2 (6.7%)	0.6
	Liver disease	2 (1.0%)	2 (1.1%)	0 (0%)	> 0.9
	Diabetes mellitus	24 (12%)	23 (13%)	1 (3.3%)	0.2
	Charlson Comorbidity index	1.0 (0.0, 2.0)	1.0 (0.0, 2.0)	2.0 (1.0, 2.8)	0.041
Laboratory values at admission	C‐reactive protein (mg/L)	150.0 (66.0, 231.3)	159.5 (67.0, 235.8)	111.5 (55.8, 221.3)	0.3
	Leukocyte count (× 10^9^/L)	13.9 (10.2, 18.3)	13.9 (10.2, 18.3)	13.2 (10.8, 19.2)	> 0.9
	Procalcitonin (μg/L)	0.5 (0.1, 4.0)	0.5 (0.1, 3.8)	2.4 (0.2, 6.6)	0.047
	SuPAR (ng/mL)	3.9 (3.3, 5.3)	3.9 (3.3, 5.3)	4.0 (3.6, 5.6)	0.6
Severity at admission	Multilobar pneumonia	47 (23%)	32 (18%)	15 (50%)	< 0.001
	Pleural effusion	37 (18%)	35 (20%)	2 (6.7%)	0.077
	CURB‐65 score				< 0.001
	CURB‐65 score 0–1	115 (56%)	111 (64%)	4 (13%)	
	CURB‐65 score 2	54 (26%)	48 (28%)	6 (20%)	
	CURB‐65 score ≥ 3	35 (18%)	15 (9%)	20 (67%)	
	Pneumonia severity index				< 0.001
	Risk class I−III	131 (64%)	122 (70%)	9 (30%)	
	Risk class IV‐V	73 (36%)	52 (30%)	21 (70%)	

*Note:* Variables are displayed as median (IQR) or *n* (%).

Abbreviation: COPD = chronic obstructive pulmonary disease.

^a^ Wilcoxon rank sum test; Pearson's chi‐squared test; Fisher's exact test.

### 3.2 Outcomes of Patients With CAP

The outcomes of the enrolled CAP patients are displayed in Table 2. The median time to clinical stability (TTCS) was longer in the patients with severe CAP compared to patients with non‐severe CAP (5.0 days vs. 3.0 days, *p* = 0.001). Length of stay was significantly shorter in the non‐severe CAP group. In the non‐severe CAP group, 86% of patients were discharged within 7 days versus 70% in the severe CAP group. There were no significant differences in 30‐day mortality, 90‐day mortality and 365‐day mortality between the two groups. A pathogen was found in 122 (59.8%) of patients. One hundred two patients (50%) had a bacterial aetiology, 20 patients (9.8%) had a viral aetiology and none of the patients had mixed bacterial/viral aetiology. Details of the microbiological results are displayed in Appendix S1.

**TABLE 2:** Outcomes of patients with CAP.

	Characteristic^a^	Non‐severe CAP, *N* = 174	Severe CAP, *N* = 30	*p*‐value^a^
Time to clinical stability	Time to clinical stability (days)	3.0 (1.0, 4.0)	5.0 (3.0, 6.0)	0.001
	Clinical stability at day 4	129 (83%)	12 (48%)	< 0.001
	Clinical stability at day 7	146 (94%)	20 (80%)	0.038
Length of stay	Length of stay (days)	5.0 (3.0, 6.0)	5.5 (4.0, 8.0)	0.019
	Discharged within 7 days	149 (86%)	21 (70%)	0.034
	Discharged within 14 days	168 (97%)	25 (83%)	0.012
Complicated course	ICU admission	0 (0%)	2 (6.7%)	0.021
	Readmission within 30 days	19 (11%)	3 (10%)	> 0.9
Mortality	30‐day mortality	4 (2.3%)	1 (3.3%)	0.6
	90‐day mortality	7 (4.0%)	2 (6.7%)	0.6
	365‐day mortality	15 (8.7%)	3 (10%)	0.7

*Note:* Variables are displayed as median (IQR) or *n* (%).

Abbreviation: ICU = intensive care unit.

^a^ Fisher's exact test; Pearson's Chi‐squared test; Wilcoxon rank sum test.

### 3.3 Relation Between Biomarkers and Risk Scores in Patients Admitted With CAP

**FIGURE 1:**
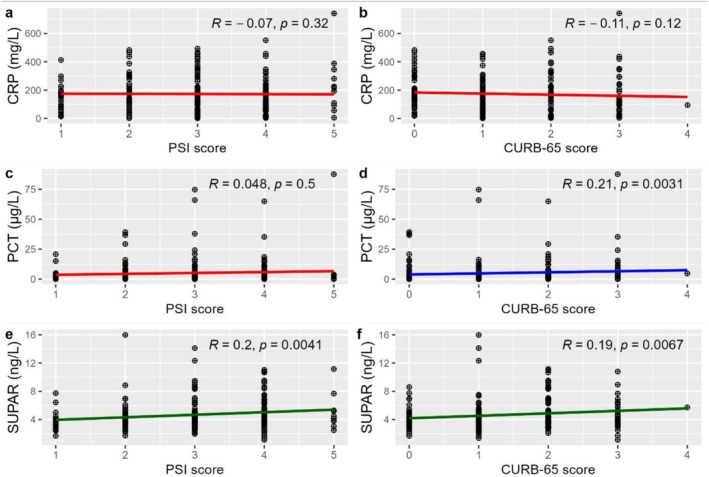
Correlation of C‐reactive protein (CRP), procalcitonin (PCT) and soluble urokinase‐type plasminogen activator receptor (suPAR) with the Pneumonia Severity Index (PSI) and CURB‐65 score.

The correlation of suPAR, PCT and CRP at admission with the PSI score and the CURB‐65 score is shown in Figure 1. SuPAR is positively correlated with the PSI score (*R* = 0.2, *p* = 0.0041) and the CURB‐65 score (*R* = 0.19, *p* = 0.0067), although the correlation was weak. PCT was positively correlated with the CURB‐65 score (*R* = 0.21, *p* = 0.0031) but was not significantly correlated with the PSI score. Levels of CRP were not significantly correlated with the PSI score or CURB‐65 score.

### 3.4 Value of suPAR in Predicting Severity of CAP

**FIGURE 2:**
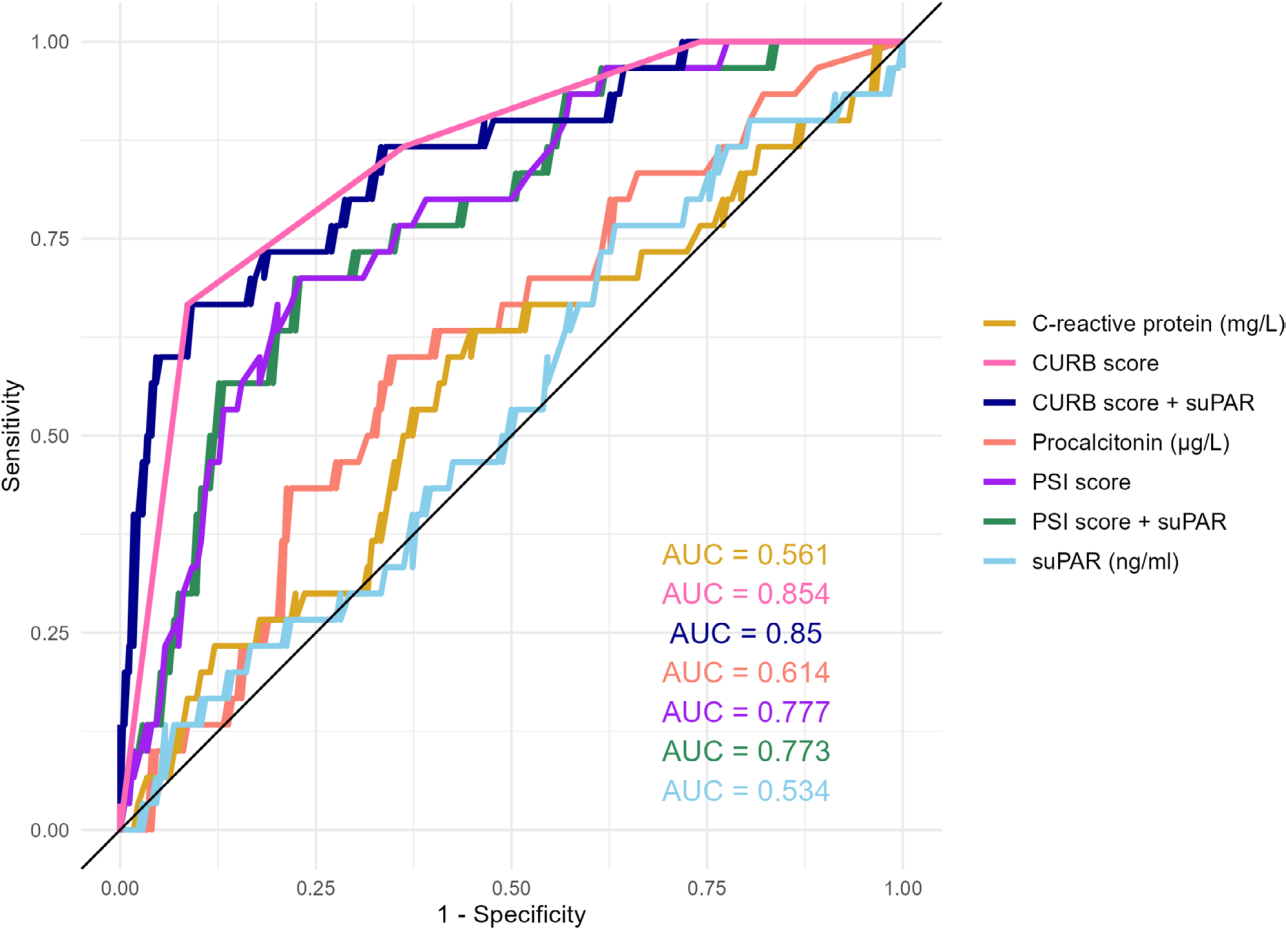
Receiver‐operating curve (ROC) of discriminating severe versus non‐severe CAP.

Table 3 displays the predictive values of PCT, CRP, suPAR, the PSI score and the CURB‐65 score for severe CAP. The ROC curve of discriminating severe versus non‐severe CAP is shown in Figure 2. A higher level of suPAR was not associated with severe CAP (OR 1.03, 95% CI 0.88–1.21). The AUC of suPAR was 0.53 (95% CI 0.43–0.64). The discriminative ability for severe CAP was similar for CRP (AUC = 0.56) and PCT (0.61), but higher for the CURB‐65 score (AUC = 0.86) and PSI score (AUC = 0.78). The likelihood ratio test of adding suPAR to the CURB‐65 score had a *p*‐value of 0.55, indicating that the model with suPAR is not better than the model with only the CURB‐65 score. Similarly, there was also no added value of suPAR to the PSI score (*p* = 0.64). Calibration showed a good model fit for the risk scores and the risk scores plus suPAR, but a poor model fit for the biomarkers alone. Internal validation using the bootstrap optimism‐corrected AUC suggested some overfitting for the biomarkers (corrected AUC for suPAR = 0.49).

**TABLE 3:** Logistic regression and ROC analysis results of prediction of severity of CAP by biomarkers and scores.

	Logistic regression			ROC analysis				
	OR	95% CI	*p*‐value	AUC	95% CI	Threshold	Sensitivity	Specificity
Procalcitonin	1.006	0.977–1.036	0.678	0.614	0.507–0.713	0.144	0.600	0.655
C‐reactive protein	0.999	0.995–1.002	0.389	0.561	0.443–0.670	0.152	0.633	0.552
suPAR	1.031	0.876–1.214	0.712	0.534	0.428–0.639	0.143	0.767	0.368
PSI score	1.038	1.021–1.055	0.000	0.777	0.687–0.858	0.167	0.700	0.770
CURB‐65 score	5.858	3.158–10.866	0.000	0.854	0.782–0.918	0.167	0.667	0.914
PSI score + suPAR	—	—	—	0.773	0.675–0.854	0.167	0.700	0.776
CURB‐65 score + suPAR	—	—	—	0.850	0.762–0.923	0.332	0.667	0.914

Abbreviations: AUC = area under the curve; CI = confidence interval; OR = odds ratio.

### 3.5 Secondary Outcomes

Detailed tables and figures of the secondary outcomes are presented in Appendix S2. Only the PSI score and the CURB‐65 score were significantly associated with TTCS at day 7 (PSI score: HR 0.80, 95% CI 0.67–0.97; CURB‐65 score: HR 0.86, 95% CI 0.74–0.99). However, for both scores, the predictive accuracy according to the AUC values was poor (PSI score: Uno's IPCW AUC = 0.58; CURB‐65: Uno's IPCW AUC = 0.57). The likelihood ratio test showed no added value of suPAR to the CURB‐65 score (*p* = 0.32) or the PSI score (*p* = 0.36). However, calibration of Cox proportional hazard model(s) showed a poor model fit for TTCS. Internal validation using the bootstrap optimism‐corrected AUC suggested some overfitting.

The PSI score, CURB‐65 score and suPAR were significantly associated with LOS at day 7. The likelihood ratio test showed that suPAR had added value to the CURB‐65 score (*p* < 0.05) and to the PSI score (*p* < 0.05). The continuous NRI for the CURB‐65 score + suPAR was 0.995, with an IDI of 0.0015. This suggests that adding suPAR improves reclassification of individuals, but the overall discriminative power of the model was only slightly improved. Similar results were seen for the model of the PSI score + suPAR, with a continuous NRI of 0.990 and an IDI of 0.0016. The Uno's IPCW AUC for the PSI score + suPAR was 0.66, and the Uno's IPCW AUC for the CURB‐65 score + suPAR was 0.63, suggesting low discriminative value. Calibration showed a moderate model fit for LOS. Internal validation using the bootstrap optimism‐corrected AUC suggested some overfitting.

Only suPAR levels were significantly associated with mortality at day 30 (HR 1.51, 95% CI 1.04–2.19). The corresponding Uno's IPCW AUC for suPAR was 0.68 (95% CI 0.51–0.86), suggesting that suPAR has moderate discriminative value for 30‐day mortality. The likelihood ratio test indicated no added value of suPAR to the CURB‐65 score (*p* = 0.08) or the PSI score (*p* = 0.08).

Assumptions were met for all cox proportional hazards models.

## 4 Discussion

In this study, we aimed to assess the prognostic value of suPAR and other biomarkers in relation to the severity of CAP. Our findings demonstrate that suPAR has limited value in predicting severe CAP as defined by the IDSA/ATS guidelines, with no significant association between higher suPAR levels and disease severity (OR 1.03, 95% CI 0.88–1.21). While suPAR exhibited limited discriminative power compared to established scoring systems like the CURB‐65 and PSI for severity, time to clinical stability and length of hospital stay, it was associated with 30‐day mortality. These findings suggest that while suPAR is not useful for predicting CAP severity, it may be useful for identifying patients at increased risk of early mortality.

SuPAR was relatively unknown as a biomarker in lower respiratory tract infections until the SAVE‐MORE trial, which showed its potential prognostic value in COVID‐19 [20]. In this trial, suPAR levels of 6 ng/mL or higher were associated with moderate to severe symptoms, suggesting that suPAR could be a useful marker in viral infections. Some studies suggested that suPAR may also have prognostic value for severity in CAP [18, 19, 23, 24], in contrast to our study. The differences between our study and previous research on suPAR's prognostic value in CAP can be explained by variations in patient populations and study designs. Wittenhagen et al. focused exclusively on patients with bacteraemic pneumococcal pneumonia, a subset with more severe infections [24]. Song et al. limited their study to patients over 65 years old. In this age group, one might expect to see lower suPAR levels due to age‐related changes in immune response [18]. Loonen et al. included only patients who had undergone a pneumococcal antigen test, creating a narrower cohort [23]. In contrast, our study had a broader CAP patient population without such specific criteria. The timing of serum sampling among different studies is different, which could explain differences in suPAR levels. Luo et al. collected serum samples during the first 2 days of admission, whereas in the present study, serum samples were directly taken at hospital admission [19].

Our findings also differ from studies in general emergency department and ICU settings where suPAR predicts severe outcomes and mortality more robustly in critically ill patients [25, 26, 27, 28]. This may be attributed to differences in patient populations, since our cohort primarily consisted of non‐severe CAP patients with a lower overall mortality rate (2.5%). In critically ill patients, suPAR reflects widespread systemic inflammation, which makes it a stronger prognostic marker. In contrast, among non‐severe CAP patients, inflammation is more localised, and suPAR's predictive value is limited. This suggests that suPAR's utility is greater in critically ill populations than in general hospitalised CAP patients. Additionally, the insights from the SAVE‐MORE trial indicate that the immune responses in viral infections may differ from that in bacterial CAP [20].

The addition of the biomarkers, CRP and PCT, did not improve prognostic performance in our study. However, findings in other studies have been mixed, with some showing added value from CRP and PCT, while others do not demonstrate a significant benefit [29, 30, 31].

This study offers insights into the prognostic role of suPAR in a cohort of hospitalised patients with CAP. By assessing multiple outcomes—such as CAP severity, length of stay, time to clinical stability and mortality—we provide a broad evaluation of suPAR's utility. The randomised controlled trial design of our present study provides reliable measurement of these outcomes, with no missing data, thereby minimising information bias.

However, there are some limitations. The overall mortality rate in our study was relatively low (2.5%). This low mortality may reflect a selection bias in the patient population due to the study design, which may not represent those with more severe CAP. Consequently, while suPAR showed a moderate ability to predict mortality, this finding requires cautious interpretation. Additionally, we did not collect data on repeated suPAR measurements during hospitalisation. This might have provided more insights into how suPAR levels change in response to treatment and disease progression. However, we observed no significant difference in suPAR levels between patients who were pretreated with antibiotics (AB) before hospital admission and those who were not (median suPAR 4.16 in pre‐treated patients vs. 3.91 in non‐pretreated, *p* = 0.70), suggesting that prior antibiotic treatment did not notably influence baseline suPAR levels. Furthermore, our study was based on available samples of an existing cohort. Our study may have been underpowered for certain secondary outcomes, such as mortality prediction. Lastly, the use of samples from a single site may limit the generalisability of our findings to other settings with different demographics or healthcare resources.

Future research should focus on evaluating suPAR's role in predicting mortality and exploring whether serial measurements over time could enhance its prognostic value. However, it is important to note that the CURB‐65 score and PSI score are already well validated and perform reliably in clinical practice and again shown in this study. Larger, multicentre prospective studies could help clarify the utility of suPAR in CAP, but their necessity should be critically evaluated.

## 5 Conclusion

In conclusion, our study demonstrates that suPAR has limited prognostic value for predicting the severity of CAP and does not offer additional benefit over established scoring systems like CURB‐65 and PSI. SuPAR demonstrates an association with 30‐day mortality, but this association may have been influenced by the low mortality rate, which in turn was due to the specific patient selection in this study. Its utility in predicting disease severity, length of stay or time to clinical stability is low. Given the good performance of CURB‐65 and PSI in assessing severity, we are not convinced that suPAR adds significant value for prognostic assessment for CAP.

## Author Contributions

L.H. contributed to study design, data analysis, data interpretation and manuscript writing. R.D. contributed to data collection and critical review of the manuscript. M.S. contributed to sample analysis and critical review of the manuscript. L.T. and W.T. contributed to study design and critical review of the manuscript. W.B. contributed to the study conception, study design, data interpretation and critical review of the manuscript.

## Conflicts of Interest

The authors declare no conflicts of interest.

## Supporting information


**Table S1** Microbiology results.
**Table S2.** Survival analysis and ROC analysis results of prediction of time to clinical stability by biomarkers and scores.
**Table S3.** Survival analysis and ROC analysis results of prediction of mortality by biomarkers and risk scores .
**Table S4.** Survival analysis and ROC analysis results of prediction of length of stay by biomarkers and risk scores.
**Figure S1.** ROC curve of prediction of time to clinical stability by biomarkers and risk scores.
**Figure S2.** ROC curve of prediction of mortality by biomarkers and risk scores.
**Figure S3.** ROC curve of prediction of length of stay by biomarkers and risk scores.

## Data Availability

The data that support the findings of this study are openly available in Baseline data REDUCE study at https://dataverse.harvard.edu/dataset.xhtml?persistentId=doi:10.7910/DVN/ZGVWLF, reference number doi:10.7910/DVN/ZGVWLF.
