# Supplementary material for: The Value of Soluble Urokinase Plasminogen Activator Receptor (suPAR) as Predictive Tool in Hospitalised Patients With Community‐Acquired Pneumonia (CAP)
Source: Clin Respir J. 2025 Jun 16;19(6):e70089. doi: 10.1111/crj.70089 (PMC12169913; doi:10.1111/crj.70089)

**SUPPLEMENTARY MATERIAL**

**Appendix 1. Microbiology results**

We defined three etiological groups based on the pathogens found by microbiological procedures.
Groups were defined as:
- viral CAP: oropharyngeal swab PCR positive for a virus and no bacteria detected. Adenoviruses, bocaviruses and rhinoviruses were not counted for this group as they are generally not considered to be virulent enough to cause CAP.
- bacterial CAP: bacteria detected by any microbial technique (blood, sputum, urinary antigen

Test or PCR) and no other pathogens identified.
- Mixed CAP: the presence of at least one bacterial species plus the presence of at least one virus (other than adenovirus and rhinovirus) by PCR, and no fungi or yeasts detected.

| **Supplementary table 1. Microbiology results** | | | |
| --- | --- | --- | --- |
|  | **Characteristic** | **Non-severe CAP**, N = 174 | **Severe CAP**, N = 30 |
| Sputum culture | Sputum culture performed | 110 (63%) | 19 (63%) |
|  | *Streptococcus pneumoniae* | 15 (8.6%) | 4 (13%) |
|  | *Moraxella catarrhalis* | 15 (8.6%) | 1 (3.3%) |
|  | *Haemophilus influenzae* | 19 (11%) | 3 (10%) |
|  | *Haemophilus parainfluenzae* | 19 (11%) | 2 (6.7%) |
|  | *Staphylococcus aureus* | 7 (4.0%) | 4 (13%) |
|  | *Pseudomonas aeruginosa* | 3 (1.7%) | 1 (3.3%) |
|  | Other | 25 (14%) | 12 (40%) |
| Blood culture | Blood culture performed | 129 (74%) | 24 (80%) |
|  | *Streptococcus pneumoniae* | 17 (9.8%) | 1 (3.3%) |
|  | *Haemophilus influenzae* | 1 (0.6%) | 0 (0%) |
|  | *Haemophilus parainfluenzae* | 0 (0%) | 0 (0%) |
|  | *Staphylococcus aureus* | 0 (0%) | 0 (0%) |
|  | *Pseudomonas aeruginosa* | 0 (0%) | 0 (0%) |
|  | Other | 6 (3.4%) | 3 (10%) |
| Oropharyngeal swab | Oropharyngeal swab performed | 170 (98%) | 30 (100%) |
|  | Adenovirus | 0 (0%) | 0 (0%) |
|  | Rhinovirus | 26 (15%) | 8 (27%) |
|  | *Chlamydia pneumophilia* | 1 (0.6%) | 0 (0%) |
|  | *Mycoplasma pneumoniae* | 8 (4.6%) | 0 (0%) |
|  | *Legionella pneumophila* | 0 (0%) | 0 (0%) |
|  | *Bordetella pertussis* | 1 (0.6%) | 0 (0%) |
|  | Human metapneumovirus | 3 (1.7%) | 1 (3.3%) |
|  | Parainfluenza viruses | 7 (4.0%) | 0 (0%) |
|  | Influenza virus A/B | 10 (5.7%) | 4 (13%) |
|  | Respiratory syncytial virus | 4 (2.3%) | 1 (3.3%) |
|  | Coronavirus | 4 (2.3%) | 1 (3.3%) |
|  | Bocavirus | 0 (0%) | 0 (0%) |
|  | Other | 2 (1.1%) | 2 (6.7%) |
| Urinary antigen test *S. pneumoniae* | Performed | 158 (91%) | 30 (100%) |
|  | Positive | 22 (13%) | 6 (20%) |
| Urinary antigen test *L. pneumophila* | Performed | 158 (91%) | 30 (100%) |
|  | Positive | 4 (2.3%) | 0 (0%) |
| All variables are displayed as n (%) | | | |

| **Appendix 2. Secondary outcomes  Supplementary table 2. Survival analysis and ROC analysis results of prediction of time to clinical stability by biomarkers and scores** | | | | | | | | | | | | |  |
| --- | --- | --- | --- | --- | --- | --- | --- | --- | --- | --- | --- | --- | --- |
|  | | **Cox Proportional Hazards Regression** | | | | | | | **ROC Analysis** | | | |  |
|  | | **HR^1^** | | **95%- CI^2^** | **P- Value** | |  | | **AUC^3^** | | **95%- CI^2^** | |  |
| Procalcitonin | | 0.976 | | 0.929-1.027 | 0.350 | |  | | 0.530 | | 0.480 – 0.580 | |  |
| C-reactive protein | | 0.984 | | 0.825-1.174 | 0.857 | |  | | 0.500 | | 0.450 – 0.550 | |  |
| suPAR | | 0.932 | | 0.807-1.056 | 0.246 | |  | | 0.540 | | 0.490 – 0.600 | |  |
| PSI score | | 0.806 | | 0.670-0.970 | 0.023 | |  | | 0.580 | | 0.530 – 0.640 | |  |
| CURB-65 score | | 0.855 | | 0.738-0.991 | 0.038 | |  | | 0.570 | | 0.520 – 0.620 | |  |
| PSI score + suPAR | | - | | - | - | |  | | 0.590 | | 0.540 – 0.640 | |  |
| CURB-65 score  + suPAR | | - | | - | - | |  | | 0.580 | | 0.530 – 0.630 | |  |
| ^1^ HR= Hazard Ratio, ^2^ CI = Confidence Interval, ^3^ AUC=Area under the curve | | | | | | | | | | | | |  |
| **Supplementary table 3. Survival analysis and ROC analysis results of prediction of mortality by biomarkers and risk scores** | | | | | | | | | | | | |  |
|  | | **Cox Proportional Hazards Regression** | | | | | | | **ROC Analysis** | | | |  |
|  | | **HR^1^** | | **95%- CI^2^** | **P- Value** | |  | | **Uno’s IPCW AUC^3^** | | **95%- CI^2^** | |  |
| Procalcitonin | | 0.482 | | 0.135 -1.716 | 0.260 | |  | | 0.650 | | 0.520 – 0.780 | |  |
| C-reactive protein | | 0.470 | | 0.157 -1.408 | 0.177 | |  | | 0.620 | | 0.470 – 0.770 | |  |
| suPAR | | 1.506 | | 1.037 -2.187 | 0.031 | |  | | 0.680 | | 0.510 – 0.860 | |  |
| PSI score | | 1.681 | | 0.786 -3.596 | 0.180 | |  | | 0.650 | | 0.490 – 0.810 | |  |
| CURB-65 score | | 1.443 | | 0.766 -2.720 | 0.257 | |  | | 0.620 | | 0.500 – 0.740 | |  |
| PSI score + suPAR | | - | | - | - | |  | | 0.720 | | 0.550 – 0.880 | |  |
| CURB-65 score  + suPAR | | - | | - | - | |  | | 0.720 | | 0.550-0.880 | |  |
| ^1^ HR= Hazard Ratio, ^2^ CI = Confidence Interval, ^3^ IPCW= Inverse Probability of Censory Weighting, AUC=Area under the curve | | | | | | | | | | | | |  |
| **Supplementary table 4. Survival analysis and ROC analysis results of prediction of length of stay by biomarkers and risk scores** | | | | | | | | | | | | | |
|  | **Cox Proportional Hazards Regression** | | | | | | | | | **ROC Analysis** | | | |
|  | **HR^1^** | | **95%- CI^2^** | | | **P- Value** | |  | | **Uno’s IPCW AUC^3^** | | **95%- CI^2^** | |
| Procalcitonin | 0.970 | | 0.924-1.017 | | | 0.207 | |  | | 0.530 | | 0.480 – 0.580 | |
| C-reactive protein | 0.947 | | 0.796-1.125 | | | 0.533 | |  | | 0.510 | | 0.460 – 0.560 | |
| suPAR | 0.784 | | 0.684-0.899 | | | 0.000 | |  | | 0.610 | | 0.560 – 0.660 | |
| PSI score | 0.702 | | 0.578-0.852 | | | 0.000 | |  | | 0.620 | | 0.580 – 0.670 | |
| CURB-65 score | 0.783 | | 0.680-0.901 | | | 0.001 | |  | | 0.590 | | 0.550 – 0.640 | |
| PSI score + suPAR | - | | - | | | - | |  | | 0.660 | | 0.610 – 0.700 | |
| CURB-65 score  + suPAR | - | | - | | | - | |  | | 0.630 | | 0.580 – 0.680 | |
| ^1^ HR= Hazard Ratio, ^2^ CI = Confidence Interval, ^3^ IPCW= Inverse Probability Censory of Weighting, AUC=Area under the curve | | | | | | | | | | | | | |

**Supplementary figure 1. ROC curve of prediction of time to clinical stability by biomarkers and risk scores**

**
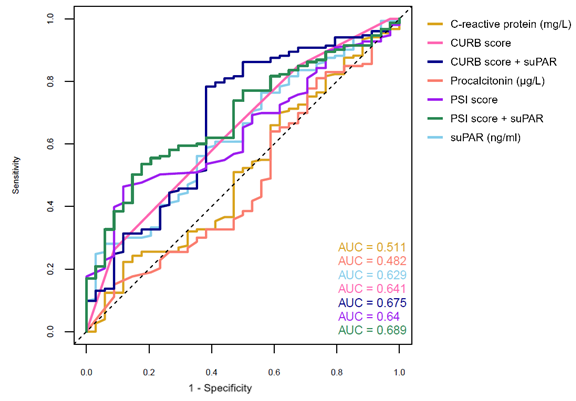

Supplementary figure 2. ROC curve of prediction of mortality by biomarkers and risk scores**


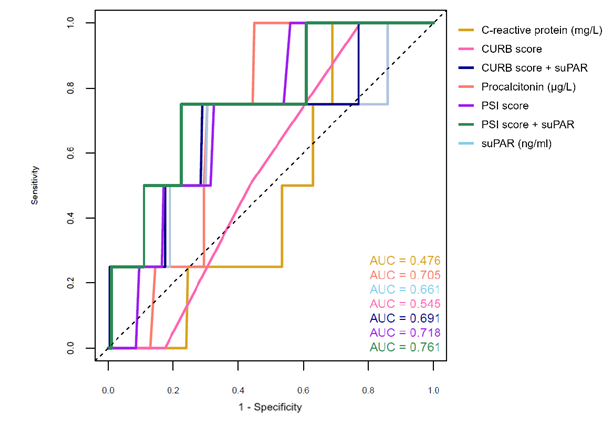


**Supplementary figure 3. ROC curve of prediction of length of stay by biomarkers and risk scores**


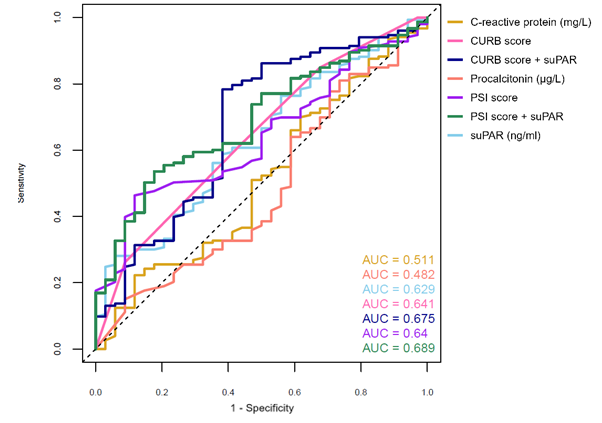

Supplement: Supplementary file 1 — Table S1 Microbiology results. Table S2. Survival analysis and ROC analysis results of prediction of time to clinical stability by biomarkers and scores. Table S3. Survival analysis and ROC analysis results of prediction of mortality by biomarkers and risk scores . Table S4. Survival analysis and ROC analysis results of prediction of length of stay by biomarkers and risk scores. Figure S1. ROC curve of prediction of time to clinical stability by biomarkers and risk scores. Figure S2. ROC curve of prediction of mortality by biomarkers and risk scores. Figure S3. ROC curve of prediction of length of stay by biomarkers and risk scores. [file CRJ-19-e70089-s001.docx]
